# Current Progress in the Development of Hepatitis B Virus Capsid Assembly Modulators: Chemical Structure, Mode-of-Action and Efficacy

**DOI:** 10.3390/molecules26247420

**Published:** 2021-12-07

**Authors:** Hyejin Kim, Chunkyu Ko, Joo-Youn Lee, Meehyein Kim

**Affiliations:** Infectious Diseases Therapeutic Research Center, Korea Research Institute of Chemical Technology (KRICT), Daejeon 34114, Korea; ckko@krict.re.kr (C.K.); leejy@krict.re.kr (J.-Y.L.)

**Keywords:** Hepatitis B virus, capsid assembly modulator, antiviral

## Abstract

Hepatitis B virus (HBV) is a major causative agent of human hepatitis. Its viral genome comprises partially double-stranded DNA, which is complexed with viral polymerase within an icosahedral capsid consisting of a dimeric core protein. Here, we describe the effects of capsid assembly modulators (CAMs) on the geometric or kinetic disruption of capsid construction and the virus life cycle. We highlight classical, early-generation CAMs such as heteroaryldihydropyrimidines, phenylpropenamides or sulfamoylbenzamides, and focus on the chemical structure and antiviral efficacy of recently identified non-classical CAMs, which consist of carboxamides, aryl ureas, bithiazoles, hydrazones, benzylpyridazinones, pyrimidines, quinolines, dyes, and antimicrobial compounds. We summarize the therapeutic efficacy of four representative classical compounds with data from clinical phase 1 studies in chronic HBV patients. Most of these compounds are in phase 2 trials, either as monotherapy or in combination with approved nucleos(t)ides drugs or other immunostimulatory molecules. As followers of the early CAMs, the therapeutic efficacy of several non-classical CAMs has been evaluated in humanized mouse models of HBV infection. It is expected that these next-generation HBV CAMs will be promising candidates for a series of extended human clinical trials.

## 1. Introduction

Hepatitis B virus (HBV) is a common, contagious liver infection that spreads from human to human through blood, body fluids, or perinatally [1]. HBV is a severe global public health burden, with particularly high endemicity levels in sub-Saharan Africa and East Asian countries [2]. Both acute and chronic HBV infections are primarily diagnosed by the serological levels of viral surface antigen (HBsAg), while acute or recently acquired HBV infections are determined by the presence of immunoglobulin M antibody to viral core antigen (IgM anti-HBcAg) [3]. Being different from this immunological diagnosis, molecular diagnosis for viral DNA is rather focused on quantification of viral genome copies, detection of drug resistant mutants, and genotyping or identification of occult HBV infections that are defined as HBV DNA-positive in the liver but HBsAg-negative [4]. Based on a survey of the seroprevalence of HBsAg, it is estimated that approximately 1.5 million people become newly infected every year. Among the 2 billion people currently infected with HBV worldwide, about 300 million are chronically infected and 820,000 die each year due to HBV-mediated liver cancer [5].

Human HBV, a member of the *Hepadnaviridae* family, is a small enveloped virus consisting of a partially double-stranded, circular DNA genome of 3.0–3.4 kb. It is classified into nine genotypes, A to I, with a putative tenth genotype, J; full-genome analysis suggests that these genotypes differ by more than 8% [6,7]. Genotypes A to D, F, H, and I are further classified into at least 35 subgenotypes [8]. A global genotypic analysis showed that the majority (96%) of chronic HBV infections are caused by genotypes C (26%), D (22%), E (18%), A (14%), and B (11%) [7], highlighting the need for effective and broad-spectrum treatment strategies.

Most people that are immunized with an HBV vaccine composed of recombinant HBsAg respond to vaccination with long-lasting serologic immunity. However, 5–10% of immunocompetent individuals do not respond to the vaccine [9,10,11], and some infants are prenatally infected by HBV-carrier mothers [12]. To reduce the risk of hepatic failure and hepatocellular carcinoma in these patients, oral nucleos(t)ide (NUC) drugs that efficiently block viral reverse transcriptase can be administered, either alone or in combination with interferon-alpha (IFN-α) [13]. Several NUCs are clinically approved, including entecavir (ETV), tenofovir, lamivudine (LAM), adefovir, and telbivudine. However, their effects are not sustained; when NUC treatment is discontinued, viral relapse and increases in alanine transaminase levels frequently occur. New antiviral strategies are in development. These aim to achieve a functional cure accompanied by the clinical outcomes of HBeAg and/or HBsAg seroconversion and removal of HBV DNA/RNA without the need for lifelong drug administration. These strategies target inhibition of the viral entry process, clearance of covalently closed circular DNA (cccDNA) using the clustered regularly interspaced short palindromic repeats (CRISPR)/Cas9 system, and interruption of the interaction between HBV X protein (HBx) and damage-specific DNA-binding protein 1 (DDB1). The degradation of viral mRNA by HBV-specific siRNA, modification of capsid assembly, or stimulation of the innate immune response with Toll-like receptor (TLR)7/8 agonists (proinflammatory cytokine activators) are additional strategies under investigation [14,15,16,17,18,19].

In this review, we will briefly introduce the HBV life cycle from the perspective of the multifaceted roles of the HBV core protein, and then summarize the classical core protein-directed antivirals termed capsid assembly modulators (referred to hereafter as CAMs), including heteroaryldihydropyrimidines (HAPs), phenylpropenamides (PPAs), and sulfamoylbenzamides (SBAs), and their mode-of-action (MOA). We will then focus on the chemical structure and antiviral efficacy of the most recently identified non-classical CAMs, and outline the current progress of clinical efficacy studies of classical CAMs, either as monotherapy or in combination with other drugs.

## 2. The HBV Life Cycle

**Figure 1:**
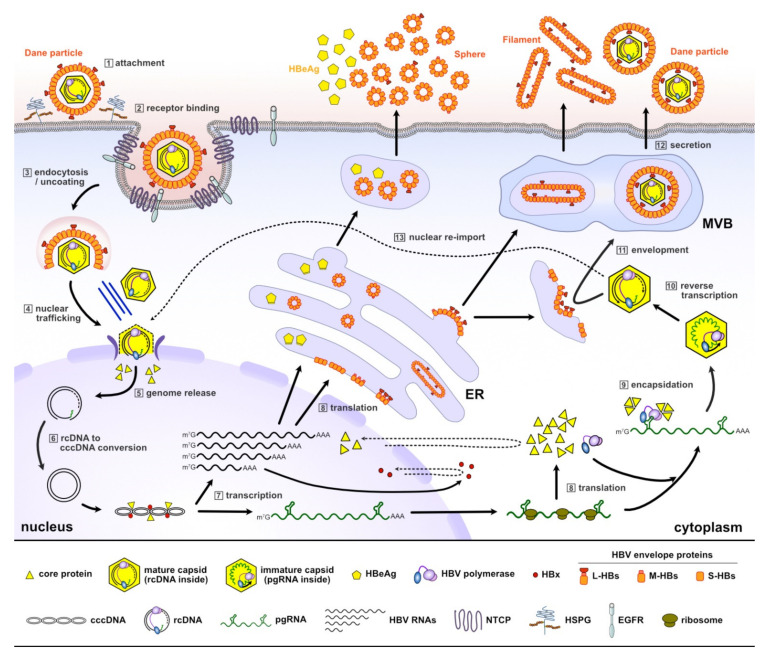
A schematic representation of the HBV life cycle. HBV enters hepatocytes by interacting with the NTCP receptor and releases the rcDNA-containing mature capsid (Steps 1–3). In the nucleus, rcDNA is converted into cccDNA, which is transcribed to generate viral RNAs (Steps 4–7). HBV pgRNA, together with HBV polymerase and core protein, are encapsulated into a newly forming capsid, inside which pgRNA is reverse-transcribed into rcDNA (Steps 8–10). The mature capsid is either coated with a host-derived lipid containing viral envelope proteins for secretion (Steps 11–12), or recycled back to the nucleus (Step 13). NTCP, sodium taurocholate co-transporting polypeptide; rcDNA, relaxed circular DNA; cccDNA, covalently closed circular DNA; pgRNA, pre-genomic RNA; ER, endoplasmic reticulum; MVB, multivesicular body; HSPG, heparan sulfate proteoglycans; EGFR, epidermal growth factor receptor.

The HBV life cycle begins with the attachment of virions called Dane particles to heparan sulfate proteoglycans on the surface of human hepatocytes (Figure 1) [20]. This initial virus–cell interaction is followed by specific engagement of the virions with a bona fide receptor, termed sodium taurocholate co-transporting polypeptide (NTCP) [21,22]. HBV virions are then internalized in a clathrin- and dynamin-dependent manner; this process is promoted by the epidermal growth factor receptor (EGFR) [23,24]. Following fusion of the virus envelope and endosome, the viral capsid, or core particle, containing partially double-stranded relaxed circular (rc) DNA is released into the cytoplasm. The capsid, which is assembled from 120 molecules of core protein dimers, protects the viral genome while it is delivered to the nucleus. In the nucleus, rcDNA is converted to cccDNA by the cellular repair machinery [25,26,27,28,29,30]. Thus, uncontrolled release of rcDNA into the cytoplasm will result in a non-viable product and may also activate the innate immune system.

HBV cccDNA is the persistent form of the virus and serves as the transcription template for all viral RNAs [31]. cccDNA is organized to a chromatin-like structure wrapped around histone and non-histone proteins, and is subjected to epigenetic regulation [32]. Two viral proteins, the core protein and HBx, associate with cccDNA [33,34,35]. While HBx plays a critical role in the cccDNA-driven transcription of HBV RNAs [36,37,38,39], the role of core protein in HBV transcription remains controversial and requires further investigation [38,40,41,42].

Hepadnaviruses replicate their genomes by reverse transcription of an RNA intermediate. As a prototypical member of the hepadnaviruses, HBV DNA synthesis is mediated by reverse transcription of a 3.5-kb HBV RNA, termed pre-genomic (pg) RNA [43]. HBV polymerase recognizes a secondary structure located near the 5′ terminus of pgRNA, termed epsilon, and the resulting pgRNA–polymerase complex is packaged into a newly assembling capsid [44,45]. Replication of the viral genome occurs exclusively within the newly forming capsid, giving rise to new rcDNA. Mutagenesis studies demonstrate that deletion or mutation of the carboxy-terminal residues of the core protein causes defects in multiple viral replication steps [46,47]. It is proposed that the HBV core protein possess nucleic acid chaperon activity, which can facilitate the structural rearrangement of the viral genome and thereby modulate viral replication [46,48]. These results suggest that capsids are not merely inert shells, but actively take part in viral genome replication.

The rcDNA-containing capsids are enveloped at the endoplasmic reticulum and released from infected cells through the multivesicular body (MVB) pathway, producing infectious Dane particles [49]. In addition to virions, an excess amount of non-infectious subviral particles (i.e., spheres and filaments composed of HBV envelope proteins (S-, M-, L-HBs)) are secreted. The mature capsid can take an alternative route to the nucleus for recycling of rcDNA to replenish the cccDNA pool [50].

## 3. The HBV Core Protein: Structure and Capsid-Forming Ability

**Figure 2:**
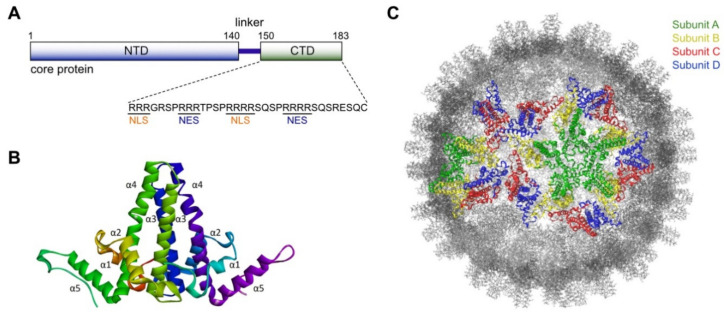
A schematic of the HBV core protein monomer, dimer and capsid. (**A**) The HBV core protein monomer is divided into three domains: an N-terminal assembly domain (NTD), a linker, and a C-terminal regulatory domain (CTD). The amino acid sequence of the CTD is shown. Nuclear localization signals (NLS) and nuclear export signals (NES) are marked below the sequence. (**B**) The X-ray crystal structure of the HBV capsid protein dimer was obtained from the Protein Data Bank (PDB ID 1QGT). Each monomer comprises five α-helices, and the α-helices are numbered. Helices α3 and α4 from opposing monomers that pack together and assemble into a four-helical bundle within the dimer. (**C**) The exterior surface of an icosahedral T = 4 capsid particle. Subunits A, B, C, and D are colored in green, yellow, red and blue, respectively.

The HBV core protein is a structural protein that plays important roles in the virus life cycle, including delivery and protection of viral genetic information, viral replication, and virion assembly. The core protein consists of 183 to 185 amino acids (depending on the genotype), and typically oligomerizes to form a T = 4 icosahedral capsid, which is composed of 120 copies of core protein dimers. The core protein can be divided into three regions: the N-terminal domain (NTD; residues 1–140), the C-terminal domain (CTD; residues 150–183), and a nine amino acid flexible linker (residues 141–149) that links the N- and C-terminal domains (Figure 2A). It is generally accepted that the NTD is sufficient and necessary for capsid assembly, while the arginine-rich CTD is involved in pgRNA packaging via its nucleic acid-binding properties [51]. The CTD contains 16 arginine residues and seven phospho-acceptor residues (six serine residues and one threonine residue) (Figure 2A), and is thus subjected to dynamic phosphorylation–dephosphorylation cycles, which are tightly regulated during virus replication [52]. Furthermore, the core protein contains both nuclear localization signals (NLS) and nuclear export signals (NES) in the arginine-repeat region of the CTD, enabling the core protein to shuttle readily between the nucleus and cytoplasm [53]. Accumulating evidence indicates that the CTD and the linker are essential for capsid assembly and viral genome replication. The NTD plus the linker (the core protein consisting of residues 1–149; Cp149) assemble spontaneously into a capsid when expressed in *E. coli*, but fail to assemble into a capsid in mammalian cells or a cell-free system using rabbit reticulocyte lysate [54,55]. This result limits the observed capsid-forming ability of Cp149 to a bacterial system. A mutagenesis study showed that the linker regulates multiple steps of HBV replication [56].

The crystal structure of the HBV core protein shows that it contains multiple alpha-helical sections [57]. The NTD of the core protein contains five α-helices: three α-helices (α1, α2, and α5) that are primarily involved in formation of a hydrophobic core, and two α-helices (α3 and α4) that are responsible for dimer formation (Figure 2B). Upon dimerization, amphipathic α3 and α4 helices from each monomer make up a four-helix bundle that protrudes from the surface of the capsid as a characteristic spike structure. The icosahedral T = 4 capsid is composed of four independent quasi-equivalent subunits (A, B, C, and D), and the resulting two types of dimer (AB and CD) display nearly identical two-fold symmetry. As shown in Figure 2C, the dimers surround the five-fold (A subunits) and quasi-six-fold (B-C-D-B-C-D) vertices.

## 4. The Mechanism of Action of CAMs

**Figure 3:**
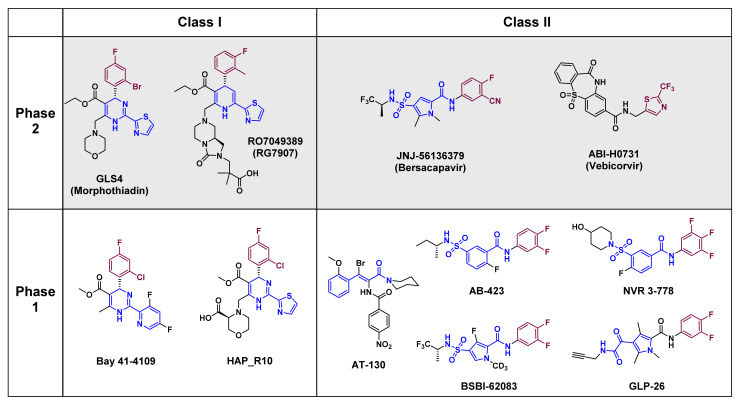
The chemical structure and clinical trial phases of classical CAMs. The core chemical skeletons are colored blue and the non-polar hydrophobic aromatic substituents that bind to the hydrophobic pocket at the interface between dimeric cores are colored purple.

**Figure 4:**
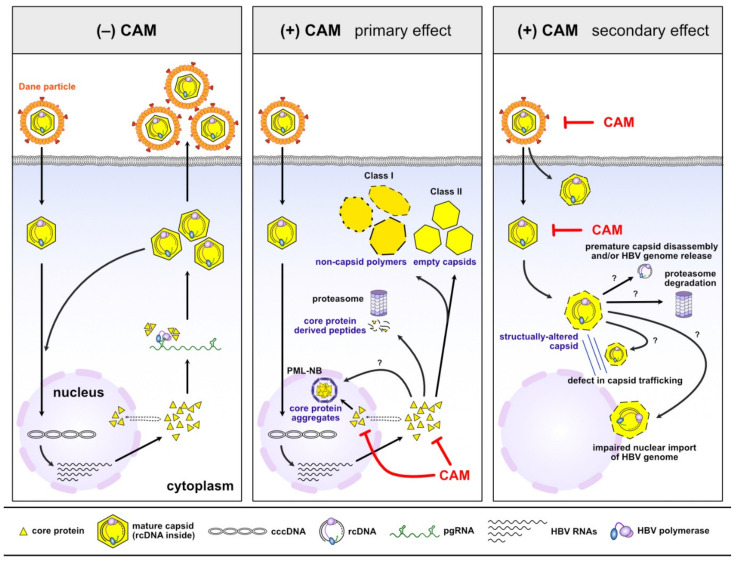
The dual effects of CAMs. In the absence of CAMs, HBV replicates in infected hepatocytes and produces progeny virions (**left**, see Figure 1 and corresponding text for details). CAMs misdirect capsid assembly and induce formation of non-capsid polymers or empty capsids depending on the CAM chemotype (**middle**). HAP-type CAMs lead to degradation and condensation of core proteins (**middle**). CAMs alter the structure and integrity of the incoming capsid and thereby prevent cccDNA formation (**right**). CAM, capsid assembly modulator; HAP, heteroaryldihydropyrimidine; PML-NB, promyelocytic leukemia nuclear bodies.

The core protein is an attractive therapeutic target due to its involvement in essential steps of the HBV life cycle (Figure 1), and since it has no known human homologs. These features have inspired development of CAMs that target the core protein by interfering with its self-assembling activity. The development of small molecules has progressed considerably over the past two decades, and some CAMs are currently under clinical investigation (discussed later in Section 6). CAMs can be grouped into two classes based on the physical effects of their treatment (Figure 3). Class I molecules, including HAPs (GLS4, RO7049389, Bay 41–4109 and HAP_R10), induce the formation of non-capsid polymers or large aggregates of core proteins [58,59], while class II molecules, including PPAs (AT-130) and SBAs (AB-423 and NVR 3–778), produce empty capsids that are devoid of pgRNA [60,61,62,63] (Figure 4; primary effect). Both classes of CAMs bind to the same hydrophobic pocket at the dimer–dimer interface [64,65,66,67] and misdirect assembly of the core protein, which eventually prevents formation of infectious progeny virions.

It is noteworthy that besides effects on capsid assembly, HAP-type CAMs appear to have additional antiviral effects in cell culture and animal models [59,68,69] (Figure 4, primary effect). Deres et al. showed that Bay 38–7690 and Bay 41–4109 deplete newly-translated core proteins via proteasome activity [68]. Although a reduction in core protein levels upon HAP treatment was independently confirmed by other research groups [59,60,62,66,69], an increase of ubiquitinated core proteins, which is a prerequisite for proteasome degradation, has not been shown. Recent studies show that Bay 38–7690 and Bay 41–4109 promote aggregation of core proteins in the nucleus [70,71], and that those aggregates are associated with promyelocytic leukemia nuclear bodies (PML-NBs) [70]. These findings bring many questions regarding the fate of the core protein upon HAP treatment, such as: (i) which pathway (degradation vs. aggregate formation) is dominant; (ii) whether those effects are dependent on experimental conditions (e.g., the concentration or duration of treatment or the cell type tested) [72]; the phosphorylation status of the core protein [73] or the chemical structure of HAPs; (iii) how a core protein that is localized in different cellular compartments (cytoplasm vs. nucleus) is targeted by HAPs; (iv) whether degradation of the core protein and/or formation of core protein aggregates is the consequence of core protein mis-assembly; and (v) whether core protein-derived peptides generated by proteasome activity are further processed and contribute to antigen presentation, i.e., whether HAP-type CAMs have immunological effects?

Several studies indicate that CAMs inhibit the establishment of infection by targeting the capsid of incoming HBV virions (Figure 4, secondary effect) [74,75,76,77,78], which is clearly different from their primary effects on capsid assembly. Considering that the HBV capsid is flexible and undergoes constant fluctuations in structure [79], it is reasonable to speculate that the CAM-binding hydrophobic pocket would become temporarily or partially accessible, which would cause CAMs to distort the structure of the intact capsid [80]. However, it is not yet fully understood how CAM-induced structural changes to the capsid affect cccDNA formation at the early stages of infection. In an effort to understand the mechanism underlying reduced cccDNA levels, Guo et al. suggested that CAMs trigger disassembly of a small proportion of mature capsids, which results in viral genome exposure or release of rcDNA from capsids [76]. However, Ko et al. suggested that structurally altered capsids have defects in capsid trafficking or release of the HBV genome into the nucleus, or that a minor proportion of capsids might be degraded in the cytoplasm [74]. Although Guo et al. and Ko et al. proposed different mechanisms for the reduction in cccDNA levels based on experimental evidence, both agreed that CAM can only mildly disrupt the mature capsids; therefore, the majority of the HBV genome remains associated with structurally altered capsids. Interestingly, CAMs can also target the capsids within HBV virions directly, and affect capsid integrity even before HBV infection [74,76], suggesting a new role of CAMs in targeting extracellular HBV particles.

Given that the HBV core protein is associated with cccDNA, CAMs could also affect cccDNA transcriptional activity [40] and stability [81], or may play a role during cccDNA formation [33], but these possibilities await further investigation.

## 5. Recent Progress in CAM Development

**Figure 5:**
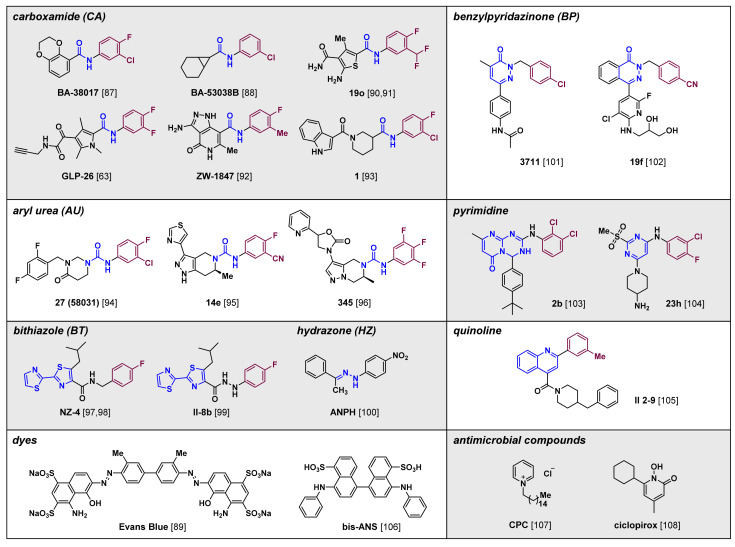
The chemical structure of non-classical CAMs, and their structure-based classifications. The core chemical skeletons are blue, and non-polar hydrophobic aromatic substituents are purple.

The chemical structure and antiviral efficacy of classic CAMs, such as derivatives of HAP, PPA and SBA, have been summarized above and in several review articles [82,83,84,85,86], so we now focus on newly developed, non-classical CAMs (Figure 5). The development of newer CAMs was prompted by the fact that despite intensive research, there are no approved CAM-based drugs, mainly due toxicity and insufficient therapeutic efficacy when used alone. As a result, several compounds have been studied and optimized structurally to provide CAMs that are new chemical entities. Remarkably, not all those compounds can be easily categorized into classes I and II, which are used for classic CAMs. They are not structurally similar to the reported scaffolds, and sometimes they display different or unprecedented functional outcomes [87,88,89]. Therefore, we aim to categorize non-classical compounds mainly by structural features rather than by MOA-based functional results.

An insight into the possible grouping of newly introduced compounds is given by comparing new CAMs with classical CAMs such as HAPs, SBAs, and PPAs. Generally, many CAM compounds bind to the dimer–dimer interface of the core protein and commonly contain halogenated aryl groups [82,83,84,85,86]. In addition, the crystal structures and results from docking studies show those aromatic fragments are mostly positioned in the hydrophobic pocket of the interface region; this is similar to the positions occupied by the aromatic fragments of HAPs, SBAs, and PPAs (Figure 3 and Figure 5, in purple). Therefore, we have made groupings by first focusing on similarities in the non-polar aromatic groups that position in the hydrophobic binding site. We then focus on common structural features of the compounds according to the linking functionalities that are attached to the aryl moieties.

### 5.1. Carboxamides

The majority of compounds discussed in this subsection are *N*-aryl carboxamides (CAs), which comprise mainly a carbocyclic or heterocyclic group at the carbonyl carbon atom and a halogenated aromatic group at the nitrogen atom of the amide. Two compounds identified from a series of carboxamides inhibit HBV replication [87,88]. Structurally, **BA-38017** (EC_50_ value, 0.16 nM) includes a fused aromatic ring in the left side of the structure, whereas **BA-53038B** contains an aliphatic fused cycle (EC_50_ value, 3.3 nM). Both compounds likely bind to the dimer interface specifically at the pocket where HAPs bind. They induced formation of morphologically normal, but empty, capsids, thereby displaying characters of class II CAMs. In addition, capsids formed in the presence of **BA-38017** and class II CAMs such as SBAs have faster mobility in an electrophoresis assay using a native agarose gel. However, **BA-53038B** caused capsids to move slower, indicating these two compounds modulate capsid assembly through different mechanisms.

More recently, the presence of heteroaromatic groups have been studied extensively to improve the potency of CA compounds. Tang and Huber et al. used thiophene as the substituent on the carbonyl moiety and disclosed the optimized compound as **19o** [90,91]. The compound is orally available and inhibits HBV DNA replication without cellular toxicity (EC_50_ value, 0.11 μM; CC_50_ value, >100 μM; bioavailability (F), 25%) (Table 1). It also induced the aggregation of core proteins in vitro, although the sizes of induced by each compound aggregated particles assessed by transmission electron microscopy analysis were different. Molecular docking studies revealed that like other CAs, the carbonyl group of the amide moiety of **19o** forms hydrogen bonds with the residual nitrogen atom of tryptophan 102 (W102). Substituents on the thiophene moiety result in additional hydrophobic and hydrophilic interactions with the core protein. In particular, the second carboxamide hydrogen bonds with the backbone carbonyl oxygen of proline 138 (P138).

The structure of **GLP-26** was released in 2019. Like **19o**, it also contains a carboxamide. Instead of the thiophene moiety present in **19o**, **GLP-26** has a methyl-substituted pyrrole and a glyoxamide as the additional carbonyl moiety at the pyrrole group, which categorizes **GLP-26** as a glyoxamide-pyrrolamide (GPA) [63]. **GLP-26** binds to the core protein and leads to formation of a smaller number of tight, firm capsid particles that resemble the morphological outcome produced by typical class II CAMs. **GLP-26** has single-digit nanomolar anti-HBV activity (EC_50_ value, 3 nM), and also decreases HBeAg secretion and cccDNA levels in vitro. Furthermore, **GLP-26** reduced HBV titers in a humanized mouse model of HBV infection, and combined treatment with ETV enhanced the efficacy of **GLP-26**. Pharmacokinetic (PK) studies in cynomolgus monkeys showed that the compound had 34% oral bioavailability and a favorable toxicity profile, thus warranting further preclinical development of this compound to potentially cure HBV infection.

Pharmacophore-based virtual exploration of various chemical structures has disclosed other carboxamide derivatives. **ZW-1847** is a new pyrazolopyridone carboxamide with moderate anti-HBV replication efficacy (EC_50_ value, 3.7 μM) [92]. An in silico structural investigation revealed that the pyrazolopyridone core occupies the hydrophobic sub-pocket and forms an additional hydrogen bond between N5-H of the core protein and the residual hydroxyl group of tyrosine 118 (Y118).

In addition to aromatic substituents at the carbonyl carbon of carboxamides, aliphatic cyclic groups have been introduced. Piperidine carboxamide derivatives were developed by high-throughput screening (HTS) and structure–activity relationship (SAR) studies [93]. The most optimized compound, compound 1, has potent anti-HBV activity in both HepAD38 and HepG2.2.15 cell lines. In addition, electron microscopy analysis showed that this compound rapidly inhibited capsid assembly without influencing morphology, thereby classifying compound 1 as a class II CAM.

### 5.2. Aryl Ureas

Aryl ureas (AUs) are important scaffolds for the development of novel CAMs. Simply, substitution of the carbon atom at the carbonyl group of carboxamides with a nitrogen atom resulted in a series of urea compounds that have anti-HBV potency based on their ability to modulate capsid assembly. For example, compound 27 is an aryl urea derived from tetrahydropyrimidinone that selectively inhibits HBV replication (EC_50_ value, 0.52 μM; CC_50_ value, 50 μM) [94]. Previously, the compound was classified as a class II CAM as it promoted assembly of empty capsids and displayed fast electrophoresis mobility in a native agarose gel.

Pyrazolopiperidine-derived AUs were identified via HTS of the Novira compound collection. An SAR study with several compounds containing heterocycles at the pyrazole moiety identified the thiazole derivative compound 14e, which has potent anti-HBV efficacy in vitro and a promising pharmacokinetic profile [95]. Compound 345 also has the urea moiety, and it contains a different fused core than compound 14e, resulting in a pyrazolopyrazine compound that has effective capsid-targeting antiviral activity (EC_50_, 0.017 μM) [96].

### 5.3. Bithiazoles

Compounds containing a tandem pair of bis-heterocycles, especially thiazoles, inhibit HBV replication. The 2,2′-bithiazole (BT) compounds **NZ-4** and **II-8b** have moderate inhibitory activity against HBV DNA replication (EC_50_ values, 1.33 μM and 2.2 μM, respectively) [97,98,99]. **NZ-4** contains a benzylic carbon atom, whereas **II-8b** contains a nitrogen atom, thereby generating the N-N bond of hydrazide in **II-8b**. **NZ-4** generates genome-free capsids without causing distinctive changes in capsid size and shape. In addition, orally administrated **NZ-4** has antiviral efficacy in duck HBV (DHBV)-infected ducks. However, despite the potential efficacy of **II-8b**, no activity on anti-HBsAg and anti-HBeAg secretion was observed. Although the binding mode of **NZ-4** has not been reported, a docking study of **II-8b** demonstrated that the fluoro-phenyl group occupies the hydrophobic pocket at the dimer–dimer interface similar to the HAP compound NVR10-001E2 [67].

### 5.4. Hydrazones

Another chemical structure, **ANPH**, containing a N-N bond was identified recently [100]. **ANPH** is an acetophenone-derived hydrazone (HZ) found by HTS using the HBV103-AdV system in Huh-7 cells (EC_50_, 0.83 μM). A detailed study revealed that **ANPH** promoted assembly of core proteins devoid of pgRNA in vitro, and that there were no distinct morphological changes compared with normal capsids. Despite representing a new structural class of CAMs, issues related to the chemical stability and toxicity of hydrazones will need to be considered during studies of further structural modifications.

### 5.5. Benzylpyridazinones

2-Benzylpyridazinones (BPs) result in formation of genome-free capsid particles. The first pyridazinone, compound 3711 promoted formation of HBV DNA-free capsids in HepG2.2.15 cells, without influencing HBV protein expression levels, the total amount of capsid protein produced, or the morphology of the resulting capsid particles [101]. Compound 3711 exhibited potent antiviral efficacy in wild-type and NUC-resistant cell lines. However, the methyl substituent on the pyridazinone core and the chloride atom in the phenyl ring were detrimental to the metabolic stability of compound 3711. Modification of compound 3711 based on SAR studies and PK improvements led to the identification of compound 19f. A ring-fusion strategy provided the phthalazinone scaffold and a pyridine group with a dihydroxy-amino pendant resulted in compound 19f, which is orally bioavailable and has high anti-HBV efficacy (EC_50_ value, 0.014 μM; F, 60.4%) [102]. Also, in compound 19f the chloride atom is replaced with a cyanide atom. The therapeutic potential of 19f was shown in a mouse model of HBV that used a recombinant adeno-associated virus carrying a replicable HBV genome.

### 5.6. Pyrimidines

Compound 2b and compound 23h are pyrimidine-based CAMs. The pyrimidotriazine compound 2b was discovered by in silico screening and has moderate inhibitory efficacy against HBV replication (EC_50_ value, 5.8 μM) [103]. Compound 23h efficiently blocks the genomic replication of HBV (EC_50_ value, 0.18 μM) [104]. Substituting the benzylic carbon atom in BPs for a nitrogen atom results in aniline-type nitrogen-based linkage to the aromatic rings that is found in both compound 2b and compound 23h. Molecular docking study of 23h proved that the halogenated aryl group is located in the interface region of the hydrophobic pocket, and that the 2-methylsulfonyl group improves binding affinity dramatically through additional hydrogen bonding with the backbone amide of L140. Compound 23h was also evaluated in uPA/SCID mice, which are a chimeric model of human liver disease. In this model, treatment with compound 23h decreased serum HBV DNA levels; furthermore, the compound acted synergistically with tenofovir.

### 5.7. Quinolines

Structure-based virtual screening suggested that quinolines could act as CAMs, and SAR studies identified the quinolyl amide **II 2-9** [105]. **II 2-9** provides a CAM with a new structure. Its activity in a bimolecular fluorescence complementation assay suggested that its mechanism of action is plausibly caused by disrupting core protein interactions.

### 5.8. Dyes and Antimicrobials

The discovery of a new use for previously developed compounds (known as repurposing) can be another way of finding new scaffolds and creating compounds with additional value. The last part of the structural summary of recent compounds in this review focuses on repurposing compounds developed for other treatments. We will highlight two dye compounds and two antimicrobial compounds.

Xiao et al. showed that a dye, **Evans blue**, inhibited HBV replication (EC_50_ value, 6.25 μM in Huh7D^hNTCP^ cells) [89]. **Evans blue** is an azo dye, used initially in vivo, that has very weak biological toxicity. Notably, two distinct host factors, NTCP and the BK_Ca_ channel, are responsible for the anti-HBV activity of the dye. Instead of binding to the core protein, the dye blocks pre-S1-mediated viral binding to host Huh7D^hNTCP^ cells through NTCP. Furthermore, **Evans blue** independently reduces the amount of calcium cations, which are essential for the assembly of the core protein. Through these mechanisms, **Evans blue** inhibits capsid formation and decreases HBV DNA levels (EC_50_ value, 12.5 μM).

Another dye, compound, 5,5′-bis[8-(phenylamino)-1-naphthalenesulfonate] (**bis ANS**), used as a fluorescent probe, binds to the core protein [106]. Although the cell-based antiviral efficacy of **bis ANS** has not been examined, the compound effectively binds to the capsid protein (E_association_, –28.0 kJ/mol). Analysis of capsid assembly by light scattering and electron microscopy showed that the compound inhibits normal capsid assembly and misdirects the formation of non-capsid polymers.

Compounds developed originally as antibacterial or antifungal agents have been repurposed as anti-HBV compounds. The antiseptic agent cetylpyridinium chloride (**CPC**) has HBV inhibitory activity, which occurs as a result of the compound rupturing and asymmetrically modifying capsid particles [107]. The antiviral activity of **CPC** was shown in a mouse HBV infection model, where it reduced HBV DNA replication in a dose-dependent manner. **CPC** and LAM had a remarkable combinatorial effect, suggesting the possible therapeutic potential of both agents.

Ciclopirox (**CPX**), a synthetic antifungal agent, has anti-HBV potency (EC_50_ value, 880 nM) that is mediated via inhibition of capsid assembly (IC_50_ value, 445 nM) [108]. **CPX** has antiviral efficacy in a humanized mouse model of liver disease (human liver chimeric uPA/SCID mice), where it acts synergistically with tenofovir.

## 6. Therapeutic Effects of Classical CAMs in Clinical Trials

According to search of progress in clinical trials of CAMs at the U.S. National Library of Medicine site (https://clinicaltrials.gov) (accessed on 10 October 2021) and the Chinese Clinical Trial Registry site (http://www.chinadrugtrials.org.cn) (accessed on 10 October 2021), a considerable number of classical CAMs are in phase 1 and 2 clinical studies (Figure 3). However, the majority of non-classical CAMs have been developed within the past five years and have not yet entered clinical trials. Here, we introduce the antiviral efficacy of presentative CAMs, including ABI-H0731, GLS4, JNJ-56136379, and RO9049389, that are in phase 2 trials, and describe combination strategies to optimize synergistic effects. This information could provide insight for the design of future clinical studies of non-classical CAMs or for determining effectiveness parameters and products based on drug combinations.

### 6.1. ABI-H7031

As a dibenzothiazepinecarboxamide derivative, ABI-H0731 (Vebicorvir) is a class II CAM. Its antiviral efficacy in humans was first investigated in a randomized, placebo-controlled phase I study in patients with HBeAg-positive or HBeAg-negative chronic HBV infection, without evidence of advanced fibrosis (NCT02908191) [109]. The participants were assigned to receive ABI-H0731 at doses of 100, 200, 300 and 400 mg, or matching placebo, once daily for 28 days. Even though 4-log reductions in HBV DNA levels were noted in the 400 mg cohort in this trial, the highest dose treatment was terminated as a grade 3 maculopapular rash occurred in a participant. From the dose-escalation study, the overall mean maximum HBV DNA reduction from baseline was 1.7, 1.9, and 2.9 log IU/mL in the 100, 200, and 300 mg cohorts, respectively. Phase 2a, multi-center, placebo-controlled studies of ABI-H0731 in combination with ETV in HBeAg-positive patients with chronic hepatitis B (CHB) (NCT03577171), or in combination with ETV and tenofovir disoproxil fumarate (TDF) or tenofovir alafenamide fumarate (TAF) in CHB patients (NCT03780543), have been completed. These trials tested ABI-H0731 and associated combinations as a finite treatment for HBV. The trial results have not yet been reported. Combination studies of ABI-H0731 with ETV and pegylated IFN-alpha (NCT04781647) or ABI-H0731 in combination with NUCs and an siRNA inhibitor (NCT04820686) of HBV are in phase 2 trials in CHB patients.

### 6.2. GLS4

GLS4 (Morphothiadin) is a dihydropyrimidine class I CAM that interferes with the assembly of the HBV core protein [110]. A randomized single-center, open-label phase 1b study (CTR20160068) was performed in China to evaluate its antiviral efficacy, pharmacokinetics, and tolerability in a combination with ritonavir (RTV; a hepatic cytochrome P450 [CYP] 3A4 inhibitor) [111]. In this study, cohorts A and B (each containing eight patients with chronic HBV infection) received GLS4 at a dose of 120 and 240 mg, respectively, combined with RTV (100 mg). Cohorts A and B and were compared with patients receiving ETV monotherapy (0.5 mg; cohort C) for 28 days. The combination used in this trial was based on the finding that addition of the CYP3A4 inhibitor enhances the serum concentration of GLS4 in humans (CTR20132137 and CTR20150230) [112]. Cohorts A and B had mean reductions in HBV DNA of 1.42 and 2.13 log IU/mL, HBsAg by 0.06 and 0.14 log IU/mL, pgRNA by 0.75 and 1.78 log copies/mL, and HBcAg by 0.23 and 0.5 log U/mL, respectively [111]. Currently, the safety, tolerability and antiviral activity of GLS4 (120 mg) are being tested in a phase 2 trial of 250 HBeAg-positive patients (NCT04147208) in combination with RTV (100 mg) and ETV (0.5 mg). This trial is also studying with ETV alone as an active comparator.

### 6.3. JNJ-56136379

The antiviral activity of JNJ-56136379 (JNJ-6379 or Bersacapavir), a heterocycle derivative of SBAs and a class II CAM, was evaluated in a double-blind, randomized, placebo-controlled phase I study (NCT02662712) [113]. Oral treatment at doses of 25, 75, 100 and 150 mg once daily for 4 weeks resulted in a reduction in mean HBV DNA levels by 1.83 and 2.16 log IU/mL from baseline at Days 15 and 29, respectively. Mean HBV RNA levels also decreased by 1.44 and 1.43 log copies/mL at Days 15 and 29, respectively. These effects were not substantially different between HBeAg-negative and HBeAg-positive patients, or between viral genotypes. The results of the phase 1 trial justified the initiation of phase 2a studies of 232 participants using longer-term dosing of JNJ-56136379 in combination with ETV or TDF (NCT03361956); the control group received NUC monotherapy. In HBsAg-positive patients, JNJ-56136379 at 75 and 250 mg (plus NUC combination) efficiently reduced mean changes in HBV DNA levels from baseline at Week 24 by 5.53 and 5.88 log IU/mL, respectively. NUC alone reduced HBV DNA by 5.21 log IU/mL. The combination treatment produced a greater decline in mean HBV RNA from baseline (by 2.82 and 3.13 log copies/mL for 75 and 250 mg, respectively) than NUC alone (a reduction of 1.43 log copies/mL). However, the effect of combination therapy on mean HBeAg changes was relatively marginal. The combination of JNJ-56136379 75 mg and NUC reduced HBeAg by 0.49 log IU/mL, the JNJ-56136379 250 mg and NUC combination reduced HBeAg by 0.67 log IU/mL, and NUC alone reduced HBeAg by 0.74 log IU/mL [114]. Phase 2 studies of JNJ-56136379 in combination with JNJ-73763989 (an siRNA therapeutic), NUCs and pegylated IFN-α 2a are ongoing (NCT04439539 and NCT04667104).

### 6.4. RO7049389

RO7049389, a class I CAM, inhibits normal capsid formation and reduces cytoplasmic levels of the core protein. In a humanized mouse model, oral administration of RO7049389 reduced serum HBV DNA significantly, as well as HBsAg and HBeAg levels [115]. Notably, treatment of CHB patients with 200 or 400 mg RO7049389 twice a day for four weeks reduced mean HBV DNA levels by 2.44 and 3.33 log IU/mL, respectively, in a phase I clinical study (NCT02952924) [116]. A phase 2 trial evaluating the antiviral efficacy and safety of this compound in multiple combinations with NUCs, siRNA (RO7445482), a TLR7 modulator (RO7020531) or pegylated IFN is underway (NCT04225715).

## 7. Conclusions

The HBV core protein is involved in almost every step of the HBV life cycle. As a multiplayer in the HBV life cycle, the core protein is an attractive target for direct-acting antivirals. CAMs are functionally classified into class I and class II; class I CAMs lead to the formation of aberrant capsids, and class II CAMs cause the assembly of pgRNA-free capsids due to rapid assembly kinetics. The chemical skeletons of CAMs are mainly divided into HAPs, SBAs and PPAs. Beyond these compounds, we focused on the chemical structure and antiviral efficacy of the most recently identified non-classical CAMs, which consist of carboxamides, aryl ureas, bithiazoles, hydrazones, benzylpyridazinones, pyrimidines, quinolines, dyes, and antimicrobial compounds. Non-classical CAMs are discriminated from earlier compounds by their chemical structure. We also highlighted the antiviral efficacy of four classical CAMs: ABI-H0731, GLS4, JNJ-56136379, and RO7049389. These drugs show considerable therapeutic effects in clinical phase 1 studies in patients with chronic HBV and have entered clinical phase 2 studies as monotherapy or in combination with approved NUCs or other immunostimulatory molecules. This review is expected to provide insights into the chemical design of additional non-classical CAMs. New chemical entities and treatment strategies for future clinical trials will achieve better therapeutic outcomes for patients with HBV.

## Figures and Tables

**Table 1:** The antiviral activity and cytotoxicity of non-classical CAMs.

Entry	Compound	Chemical Class	EC_50_ Value (μM) ^a^	CC_50_ Value (μM) ^b^	Cell Line	Ref.
**1**	**BA-38017**	CA	0.16	>50	AML12HBV10	[87,88]
**2**	**BA-53038B**	CA	3.3	>100	AML12HBV10	[87,88]
**3**	**19o**	CA	0.11	>100	HepAD38	[90,91]
**4**	**GLP-26**	CA	0.003	>100	HepAD38	[63]
**5**	**ZW-1847**	CA	3.7	>100	Huh-7	[92]
**6**	**1**	CA	0.02	>30	HepAD38	[93]
**7**	**27 (58031)**	AU	0.52	>50	AML12HBV10	[94]
**8**	**14e**	AU	0.012	n.d.	HepG2.2.15	[95]
**9**	**345**	AU	0.017	n.d.	HepG2.2.15	[96]
**10**	**NZ-4**	BT	1.33	>50	HepG2.2.15	[97,98,99]
**11**	**II-8b**	BT	2.2	>50	HepG2.2.15	[97,98,99]
**12**	**ANPH**	HZ	0.83	>100	Huh-7	[100]
**13**	**3711**	BP	1.5	>100	HepG2.2.15	[101]
**14**	**19f**	BP	0.014	>100	HepG2.2.15	[102]
**15**	**2b**	pyrimidine	5.8	>100	HepG2.2.15.7	[103]
**16**	**23h**	pyrimidine	0.18	n.d.	Huh-7	[104]
**17**	**II2-9**	quinoline	1.8	>20	HepG2.2.15	[105]
**18**	**Evans blue**	repurposed	6.25	>100	Huh7D^hNTCP^	[89]
**19**	**bis ANS**	repurposed	n.d.	n.d.	-	[106]
**20**	**CPC**	repurposed	n.d.	n.d.	-	[107]
**21**	**ciclopirox**	repurposed	0.88	n.d.	HepG2.2.15	[108]

^a^ Fifty percent of the maximum effective concentration, determined by measuring the reduction of viral DNA replication using PCR. ^b^ Fifty percent of the maximum cytotoxic concentration, determined by cell viability tests. Abbreviations: CA, carboxamide; AU, aryl urea; BT, biothiazole; HZ, hydrazone; BP, benzylpyridazinone; n.d., not determined.
